# Design of a low-cost mobile multispectral albedometer with geopositioning and absolute orientation

**DOI:** 10.1016/j.ohx.2022.e00324

**Published:** 2022-06-10

**Authors:** J.S. Botero-Valencia, M. Mejia-Herrera, Joshua M. Pearce

**Affiliations:** a Grupo de Sistemas de Control y Robótica, Engineering Faculty, Instituto Tecnológico Metropolitano, Medellín, Colombia; b Department of Electrical & Computer Engineering, Ivey Business School, Western University, London, ON, Canada

**Keywords:** Absolute orientation, albedometer, geopositioning, multispectral measurement, photovoltaic energy, reflectivity

## Abstract

Albedo is the percentage of radiation that a given surface reflects. Its study is important to evaluate thermal effects in buildings, generation capacity with bifacial panels, among others. In this work, the design and validation of a low-cost mobile albedometer is presented, which measures the reflection in 8 spectral bands in the visible, additionally the system is equipped with a Global Navigation Satellite System (GNSS) receiver, to reference its position and an Inertial Measurement Unit (IMU) to know its absolute orientation, make corrections in real time or detect errors. The purpose of designing the mobile device is to measure a larger area and, since it is georeferenced, it is to feed GIS tools that allow designers to use the information.

## Graphical abstract

**Figure:**
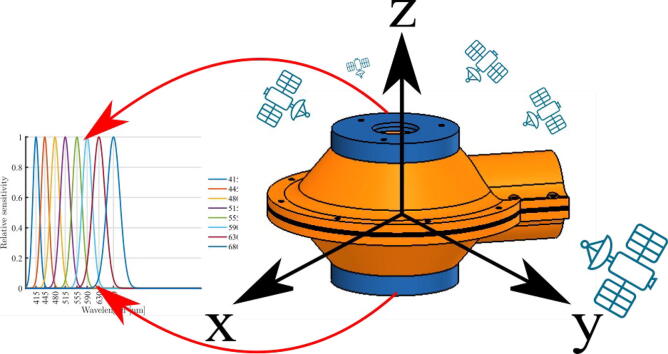

**Specifications table:**

**Table:** 

**Hardware name**	Open source wireless geopositioning albedometer.	
**Subject area**	• Engineering • Instrumentation • Internet of things	
**Hardware type**	• Measuring physical properties and in-lab sensors • Field measurements and sensors • Electrical engineering and computer science	
**Open source license**	Creative Commons Attribution-ShareAlike license	
**Cost of hardware**	USD 179.24	
**Source file repository**	https://doi.org/10.17605/OSF.IO/BVJH2	

## Hardware in context

**Fig. 1:**
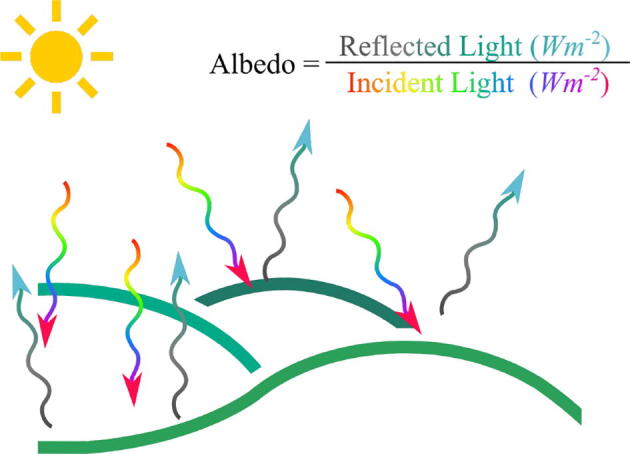
Albedo.

Albedometers measure albedo (solar reflectance), which is the ratio of the reflected to the global solar radiation. The albedo is a measure of 0 to 1 that represents the ratio between the light radiation emitted by an object and the radiation it receives, as seen in the Fig. 1.

Generally, albedometers are composed of two pyranometers where one faces up and measures the global solar radiation, and the downfacing one measures the reflected solar radiation. Albedometers are most often used for general meteorological observations as part of a meteorological station [1], [2], building physics [3], [4], pavement characterization [5], roof [6] and roof reflectance studies for aging [7]. Albedometers are also used for climate studies including glaciers [8], snow and ice [9] and solar collector testing for thermal and photovoltaic (PV) systems [10]. Albedo measurements have become increasingly important for bifacial solar PV systems [11], [12], [13], [14], [15]. Albedometers are also increasingly important for three new solar PV applications: i) albedo optimization for low concentration PV systems [16], [17], [18], [19], [20], [21], ii) agrivoltaic crop albedo [22], [23], and iii) PV material selection for optimization of a given location [24], [25]. As there are so many varied applications for albedo measurements there is a wide range of commercial developments [4], [26], [27], [28], which use pyranometers to obtain albedo data, in addition to the use of remote sensing and satellites [29]. Such systems, however, are usually expensive and might require specific equipment depending on the brand [30], [4], with prices between 600–2000 USD making it difficult to deploy or use with other technologies. And some of these systems are not re-programmable and cannot be adapted to other specific tasks for research purposes.

For that reason, low-cost albedometers can be found in the literature. A system with two *Hamatsu* micro spectrometers for the measurement of albedo from an unmanned aircraft system have been developed [31]. The obtained results are compared with the information provided by satellite systems (MODIS and LANDSAT17). In [32] the pyranometer that is oriented towards the study surface is protected with a barrier that allows reducing the area of interest to obtain detailed measurements and that is not affected by the radiation of surrounding objects the results obtained demonstrate a low variation (0.005) with respect to the ASTME1918 standard. In [33] a system is designed that uses multiple interference filters and a silicone detector to take albedo measurements. The interference filters used are customized with the MODIS spectral response band to obtain comparable measurement ranges. A low-cost easily upgradable and customizable albedometer is still needed. One approach that has consistently reduced the costs of scientific hardware [34], [35], [36], [37], [38], while improving customization [39] and quality is the use of open hardware [40]. These properties make science more accessible to a greater number of scientists particularly those in low resource settings [41], [42] and the developing world [43], [44]. To leverage these advantages and benefits of open hardware [45], [46], in this study an open source design of a low cost mobile multispectral albedometer with geopositioning and absolute orientation systems is provided, built, tested, and validated.

## Hardware description

The proposed device is a geo-positioned low-cost albedometer, that allows attitude correction of multispectral sensors using data from absolute position sensors. In order to enable future customization, the construction of the system was carried out using additive manufacturing (AM) [47] and computer-aided design (CAD) [48]. All of the design files including the bill of materials can be found at osf.io/th8y7 and are under the following open hardware licenses: CC BY 4.0.

**Fig. 2:**
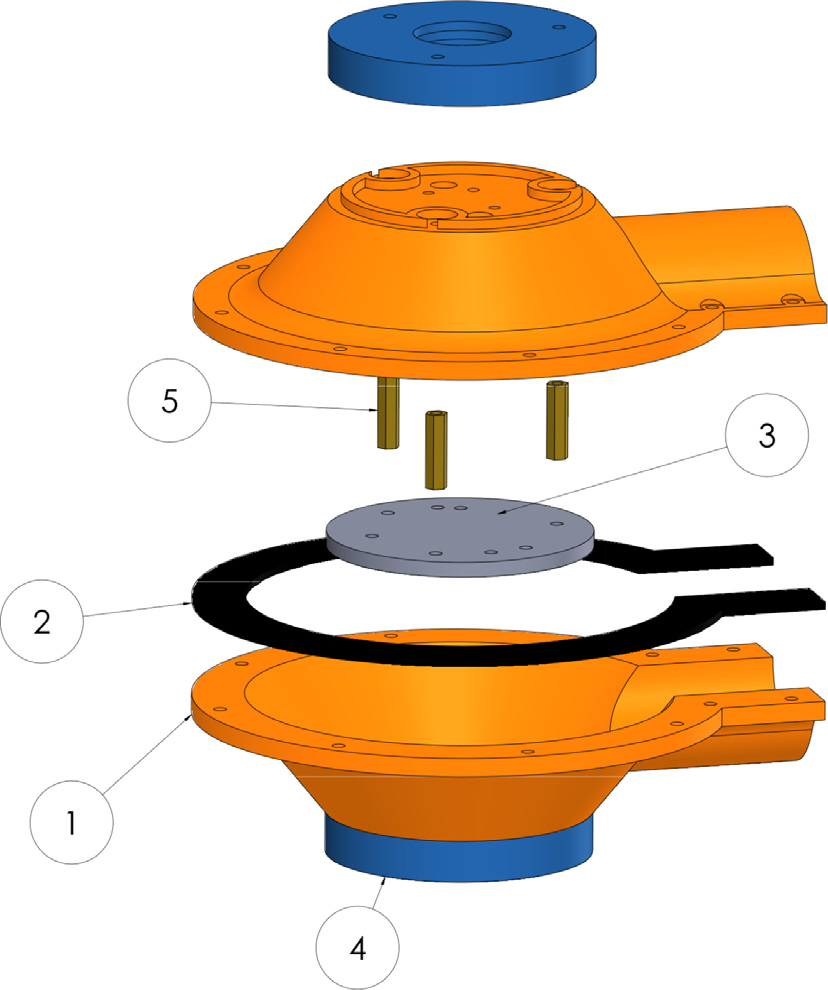
Exploded assembly of the proposed albedo-meter system.

The system was strengthened for outdoor use by implementing o-rings and silicon to reduce joints water filtration. Nevertheless, the system ingress protection level has not been determined yet. The proposed system consists of two identical plates shaped like a hollow truncated cone (1) with an extrusion for a one inch tube that facilitates its installation. The plates have centered perforations in which a dome-shaped diffuser is placed that facilitate the collection of radiation, and is secured using an additional piece (4) as seen in Fig. 2.

The proposed hardware provides several advantages:

• Georeferencing of albedo allows measurement over large areas, integration with GNSS makes it possible to move and acquire at the same time.
• Multispectral sensors allow to differentiate the reflection in bands, so you can know more specifically the reflection and even know the color of the surface.
• The use of an IMU allows the absolute angle to be corrected or known so that errors are detected in the acquisition process or corrections are made.
• Mixing geographic coordinates with multispectral albedo acquisition allows the construction of interpolated maps for each band.
• SD memory integrated into the system allows to store large amounts of data at high speed and to do the process offline.

## Design files

**Fig. 3:**
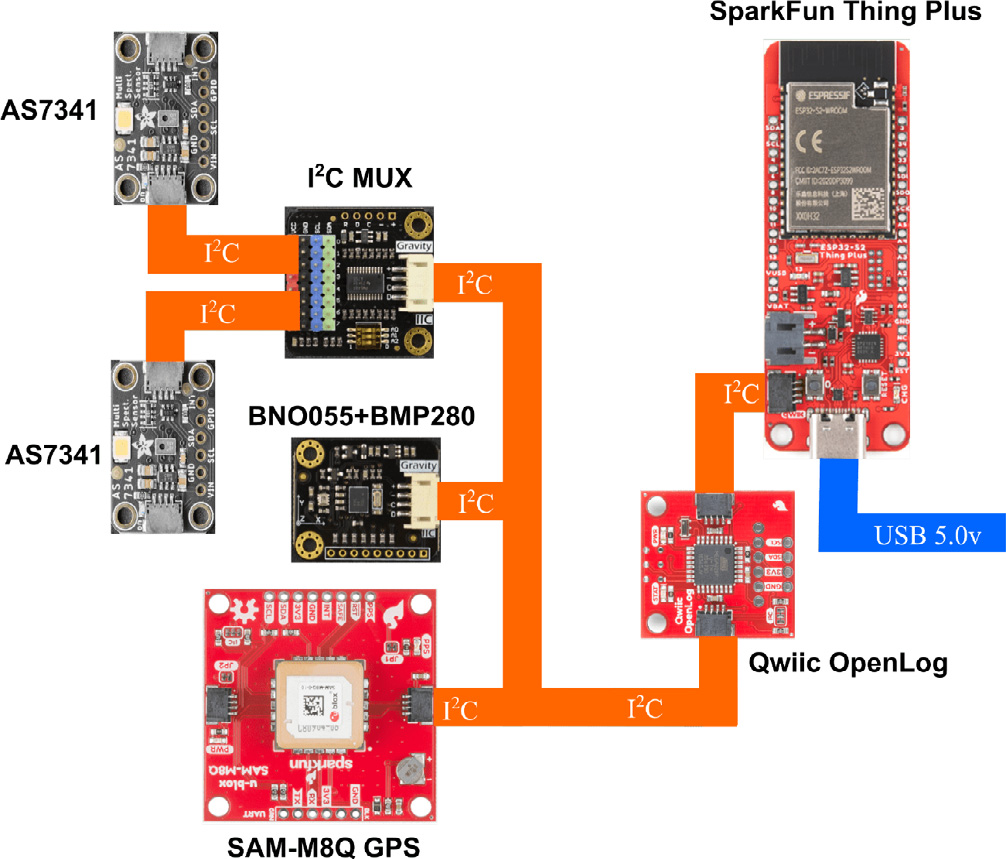
Connection diagram of the designed system.

The components were 3-D printed out of acrylonitrile styrene acrylate (ASA) and thermoplastic polyurethane (TPU) on an open source Ender 5 Pro (Creality) 3-D printer using the following settings: ASA pieces, 30% of Hexagonal Infill, bed temperature 90°C, extruder temperature 250°C and TPU (Gasket), 40% of Hexagonal Infill, bed 50°C and extruder 220°C. The system is symmetrical and allows the acquisition of light radiation in opposite directions of the same axis. The plates are joined using screws and M3 separators. In the middle of the plates, a custom designed and 3-D printable TPU Gasket (2) is located to increase protection against water. Inside the system are: Two ten-channel AS7431 [49] visible radiation sensors connected to a MUX I2C, which are used for radiation measurement and albedo estimation fixed to (3) and fixed using brass M3 female–female separators (5). A sensor [50]: BNO055 of absolute positioning of nine degrees of freedom that calculates the angle of the acquisition to make future corrections or light decomposition. A SAM-M8Q GPS [51] with multiband reception that allows positioning with much lower error, to relate the acquired data to a geographical position, and allow the creation of albedo maps or other geolocated studies. All these devices are connected via I2C to an OpenLog which acts as a black box to store the collected information into an SD memory and connects sensors to a Sparkfun Thing Plus [52] for data collection and energy management as shown in Fig. 3. The current consumption of the components is approximately: 30 mA for the SAM-M8Q, 12 mA for the OpenLog, 5 mA for each of the AS7431, 5 mA for the BNO055, and 80 mA for the ESP32, for a total of 137 mA.

### Design Files Summary

This section describes each of the files necessary for the construction of this project that can be manufactured using 3D printing. The Table 1 below also indicates its location, all files are available in both STL and STEP formats.

• AlbedoBottom, It is the main part, two equal pieces are joined together, inside is the electronics and sensors.
• AlbedoTop, It is the piece that holds the Dome that protects the multispectral sensors, it must be printed twice.
• Dome, The dome is the cover of the multispectral sensors, it can be printed on transparent polycarbonate.
• Gasket, It is a piece that serves to prevent water from entering, it is printed in TPU. Being a piece it allows to disassemble the system.
• Support, It is the internal part where the Multiplexer I2C, the IMU and the GPS must be assembled, these are fixed internally to the Albedo Bottom.

**Table 1:** Design Files.

**Design file name**	**Open source license**	**Location of the file**
AlbedoBottom (.step.stl)	GNU GPL v3.	https://osf.io/wzqy6// https://osf.io/p5jm4/
AlbedoTop (.step.stl)	GNU GPL v3.	https://osf.io/hdgkv// https://osf.io/m5cu2/
Dome (.step.stl)	GNU GPL v3.	https://osf.io/fu6ek// https://osf.io/9ujsa/
Gasket (.step.stl)	GNU GPL v3.	https://osf.io/cqx2s// https://osf.io/yzdn9/
Support (.step.stl)	GNU GPL v3.	https://osf.io/5whp2// https://osf.io/cjnre/

## Bill of materials

This section presents a list of the parts that must be purchased for the manufacture of this device, including the current cost and a possible supplier. The use of ASA as a printing material can be reviewed at [53]. The list of materials can be found in Table 2.

**Table 2:** Bill of materials

**Designator**	**Component**	**Qty**	**Unit cost**	**Total cost**	**Source of material**
ESP32 Thing Plus	MCU	1	$22.50	$22.50	t.ly/VuRG
AS7341	Sensor	2	$15.95	$31.90	t.ly/0Lxi
BNO055	Sensor	1	$25.90	$25.90	t.ly/14Ps
Mux I2C	Interface	1	$6.90	$6.90	t.ly/ALPy
SAM M8Q	GPS	1	$42.95	$42.95	t.ly/lVw9
Qwiic OpenLog	SD log	1	$18.50	$18.50	t.ly/ZhJh
Qwiic cable	Interface	6	$1.60	$9.60	t.ly/5KrP
ASA Filament	Structural	0.5	$29.99	$15.00	t.ly/hy8I
TPU Filament	Structural	0.1	$29.99	$3.00	t.ly/ZHb7
PC Filament	Structural	0.1	$29.99	$3.00	t.ly/fz75

				$179.24	Total

## Build instructions

**Fig. 4:**
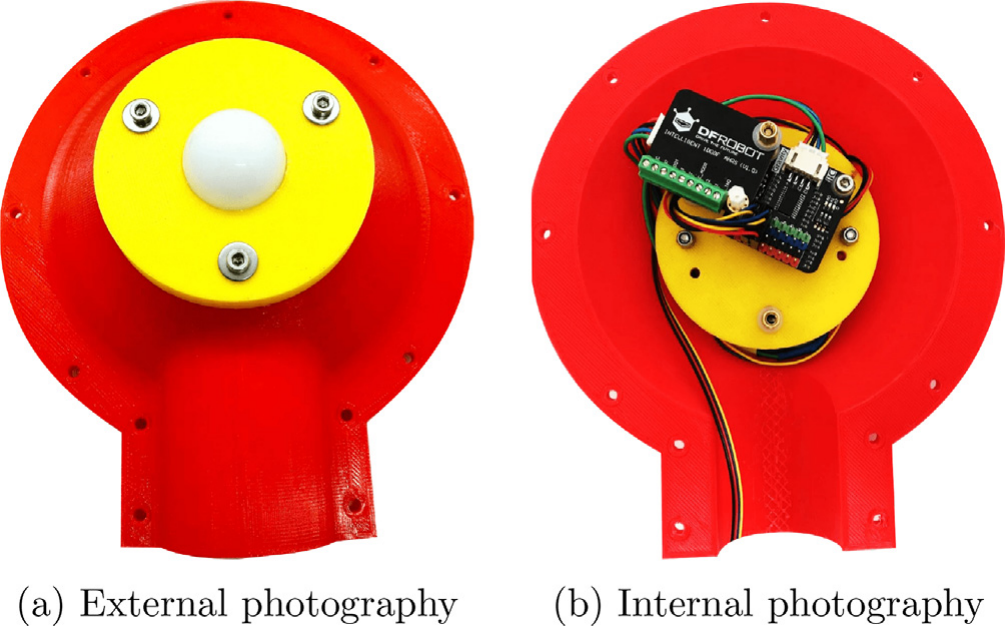
Photographs of the assembled device.

The proposed design was 3-D printed and painted and assembled as shown in Fig. 4. In Fig. 4a, the external face is shown, the white component is the diffuser coating that directs the light to the AS7341, two equal parts of this piece are assembled, It can also be printed on transparent polycarbonate (Dome.stl in Table 1), its appearance is white, but its transmittance is close to 70%. In Fig. 4b, the internal face is shown, so that the multiplexer can be seen for the connection of the two AS7341 components (that have the same I2C address) and the BNO055. The GPS is fixed on the opposite side of the yellow component. The MCU (ESP32 Thing), connects outside of this piece, the space that remains between the two assembled faces is for a 1-inch PVC pipe for mounting. For the electronic connection of the components, the schematic shown in Fig. 3 can be taken into account. For the mechanical assembly, can be guided of Fig. 2. All screws and spacers used are M3.

## Operation instructions

After having the system completely assembled, and confirming that the program is loaded in the MCU, it will return the connection startup data, and the output shown in 1 should be presented, this information is also stored in the SD memory with the file name “SYSFN.txt”, this output is included in the code to facilitate the debugging to the users, and is the status resulting from the scanning and enabling of the sensors connected to the I2C bus and the multiplexer. The OK status of each sensor indicates that it is connected and functional and ER that it is disconnected or had a problem. The sampling interval can be set using the ${\mathrm{READ}}_{D}\mathrm{ELAY}$ variable (in milliseconds) in the code.

**Table:** 

**Listing 1**: Output at startup
[000000000036][000000000036][SYST] INI: Begin OK
[000000000570][000000000570][BN55] INI: Begin OK
[000000000753][000000000753][AS41] INI: Begin OK
[000000000878][000000000878][AS42] INI: Begin OK
[000000001007][000000001007][SM8Q] INI: Begin OK
[000000001137][000000001137][SYST] STF: Begin OK

After the acquisition starts, and with the sampling interval defined, the data is shown on the MCU output and stored in the SD memory. Four files are generated for the multispectral sensor information, “AS411AVI.txt” and “AS412AVI.txt” correspond to one of the multispectral sensors and “AS413AVI.txt” and “AS414AVI.txt” to the other. Two files are also generated to store the IMU information “BNO55DA0.txt” and “BNO55DA1.txt”, and finally, a file to store the GNSS information called “SAM8QGPS.txt”. It is important to highlight that the PDOP (3D Dilution Of Precision) and the SIV parameter (Number of satellites used in fix) are stored, in order to know in the acquisition process, with what precision and under what conditions the GPS coordinates were saved. Listing 2 shows the headers of these files.

**Table:** 

**Listing 2**: Output at startup
AS411AVI.txt -> 415 445 480 515 CLR NIR
AS412AVI.txt -> 555 590 630 680 CLR NIR
AS413AVI.txt -> 415 445 480 515 CLR NIR
AS414AVI.txt -> 555 590 630 680 CLR NIR
BNO55DA0.txt -> ACX ACY ACZ MAX MAY MAZ
BNO55DA1.txt -> GYX GYY GYZ EUX EUY EUZ
SAM8QGPS.txt -> SIV LON LAT ALT DOP HEA

Finally, to reconstruct the information as a heat map, it is recommended to execute the following procedure tested in QGIS 3.22.3:

• Create a CSV file using the headers “Lat”, “Lon”, and “Z”, where the coordinates of the file should be copied, the Z column should be calculated by dividing the sum of each row of the values of the files “AS411AVI.txt” and “AS412AVI.txt”, between “AS413AVI.txt” and “AS414AVI.txt” respectively, to get the total reflectance value in this case.
• Add an Open Street Map layer in the menu, Layer -> Add Layer -> Add XYZ Layer. If not available use the URL: http://tile.openstreetmap.org/z/x/y.png
• Add the datapoints, using the menu Layer -> Add Layer -> Delimited Text Layer, add the previously created file, having selected the option “Geometry GRS EPSG:4326-WGS 84”. The datapoints should appear on the Open Street Map layer.
• Finally, using the Processing Toolbox -> Mesh -> TIN Mesh creation, interpolation can be generated with the loaded information. The maximum and minimum value, the color ramp, among others, can be configured.

## Validation and characterization

**Fig. 5:**
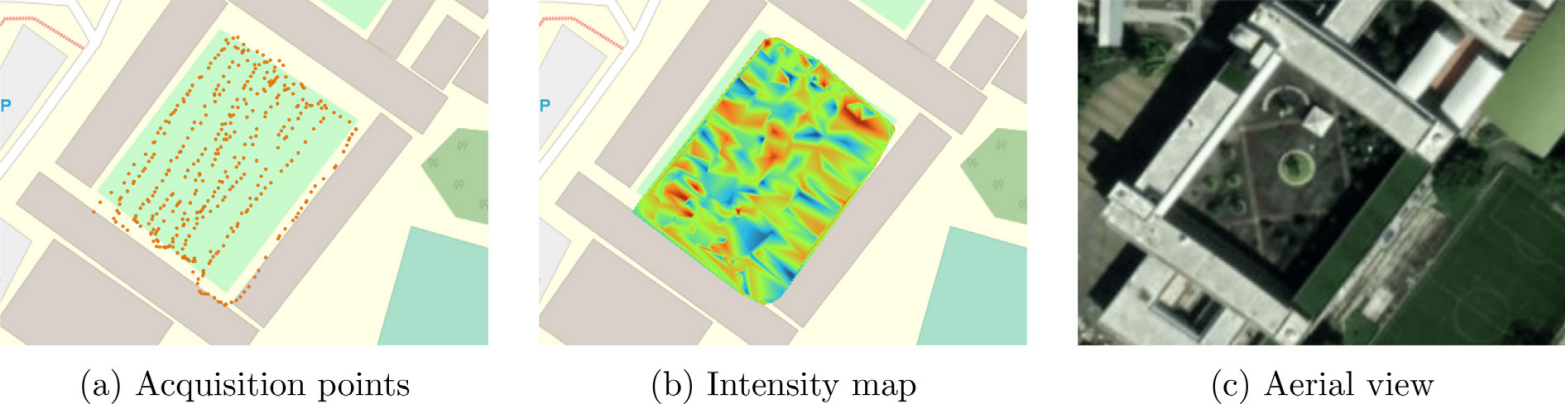
Gathered information Maps.

To show the utility of the georeferenced system, it was mounted on a mobile platform and moved through the central square of the campus at the Instituto Tecnológico Metropolitano in Medellín-Colombia, the data was acquired at noon. In Fig. 5a, the coordinates where samples were taken are observable and Fig. 5b shows the result after processing the data in QGIS and performing a Triangulated Irregular Networks (TIN) interpolation. In this case a resolution of 200 pixels is used. This application makes it easy to estimate albedo over large areas. The albedo in this case is between 0.09 and 0.14, and the ground type is concrete.

**Fig. 6:**
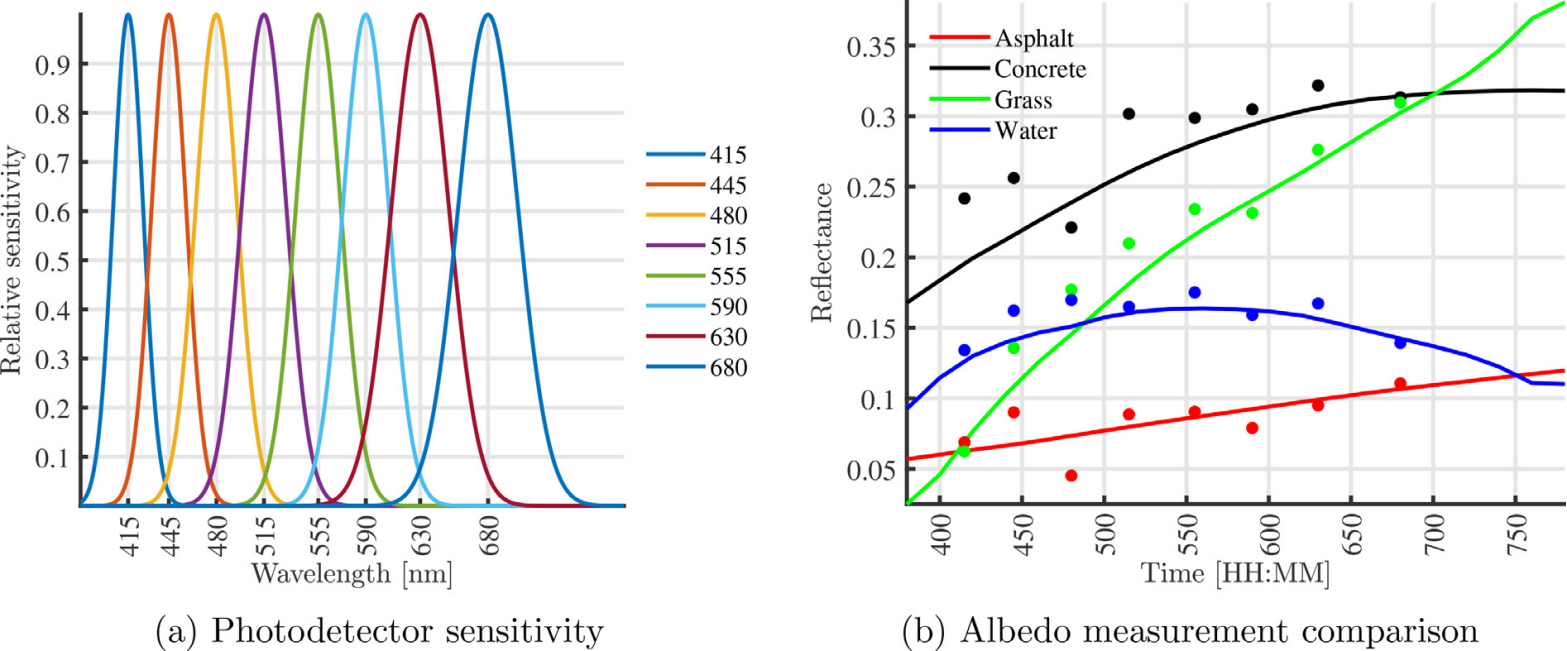
Multispectral albedo measurement.

Finally, to show the usefulness of using a multispectral photodetector, a specific experiment was carried out in which the reflectance of typical surfaces such as asphalt, concrete, grass and water was acquired with a reference spectrometer and the albedo was estimated with the proposed device. The gain of the bands must be adjusted using a method like the one presented in [54], or using a reference blank. In Fig. 6a, the sensitivity of the sensor bands used in this work (AS7341) are presented and in Fig. 6b, the reflectance acquired with an OHSP-350 spectrometer is shown in solid lines and the albedo markers for each one of the bands available on the AS7341. The maximum reflectance error of our system with respect to the reference spectrometer is 3%, the reference equipment has an accuracy of 1.5% with a resolution of 0.2 nm.

## Discussion and future work

The presented work presents the combination of different measures that are useful to perform albedo characterization on surfaces in a way that can be georeferenced and consider a measure in different spectral bands, this has important advantages such as being able to develop maps using carts, robots or other ways in which the proposed system can be moved while the acquisition is done, the reflectance in bands can have an important utility if an approximation of the color of the surface is to be known. The proposed system has a lower cost (180 USD) than the typical implementation of using two pyranometers (800 USD), and has the advantage of being georeferenced, having the IMU to ensure parallel to the ground, and having different reference spectral bands. The price and integration of the other measurements also facilitates the scalability and blending of this application with solar PV projects so that this measurement can be considered in the development of bifacial solar farms where the measurement is taken at more than one point.

The most important limitation of the proposed system is related to the use of a standard GPS that can reach according to the manufacturer an accuracy of 2.5 m, but in field conditions is approximately 5 m. This limits the meshing that could be obtained from a surface, however, this can be easily replaced by a GPS RTK to increase the accuracy to 0.025 m, although its price can be 6 times higher than the standard GPS. Another limitation to consider is the speed, the refresh rate of the GPS in the best case is 1 Hz, so it is recommended that for the acquisition in motion, pauses are made to fix the acquisition, this pause state can be verified with the information stored by the IMU. As future work, it is important to consider a sensor or array of distance sensors that would also allow consideration of this measurement and the integration of at least ambient humidity that may affect reflectance.

The proposed system enables measurement of the albedo in different spectral bands while simultaneously acquire the GPS coordinates, and the orientation with respect to the ground using an Inertial Measurement Unit. The combination of these data enables users to build maps of albedo intensity in large surfaces automatically.

Open hardware initiatives are very important to accelerate the advancement of science and its applications in engineering, in this case, with the proposed system, it is expected that it can be applied to characterize large areas of land and that this measure will be useful to assess the estimated viability or efficiency that could be had with the installation of bifacial solar panels.

## Human and animal rights

No human or animal studies were conducted in this work.

## Declaration of Competing Interest

The authors declare that they have no known competing financial interests or personal relationships that could have appeared to influence the work reported in this paper.
